# Condition dependence of the multimodal courtship of a parasitoid wasp (*Leptopilina heterotoma*)

**DOI:** 10.1093/beheco/arag077

**Published:** 2026-08-06

**Authors:** Janka Plate, Taina Conrad, Johannes Stökl

**Affiliations:** Department of Evolutionary Animal Ecology, University of Bayreuth, Universitätsstr. 30, Bayreuth 95447, Germany; Department of Evolutionary Animal Ecology, University of Bayreuth, Universitätsstr. 30, Bayreuth 95447, Germany; Slovenian Academy of Sciences and Arts Ljubljana, Institute of Biology, Novi trg 2, 1000 Ljubljana, Slovenia; Department of Evolutionary Animal Ecology, University of Bayreuth, Universitätsstr. 30, Bayreuth 95447, Germany

**Keywords:** insect communication, sexual selection, mate choice, signal content, hymenoptera

## Abstract

Courtship displays often consist of more than 1 modality, despite additional costs associated with multiple modalities. Because courtship signals under strong sexual selection often show heightened condition dependence, they may convey information about mate quality. However, much of the research on condition dependence has focused on individual modalities. Theoretically, different modalities within 1 multimodal signal could show different degrees of condition dependence, depending on how strong the selection pressure is on each modality. Here, we investigate how male condition affects the multimodal courtship of the parasitoid wasp *Leptopilina heterotoma*. Specifically, we tested how 2 components of the multimodal courtship*—*a chemical signal (cuticular hydrocarbons, CHCs) and a vibratory signal (wing fanning)—are affected by age and nutritional state. We found that the 2 modalities responded differently to the male condition: Wing fanning was affected by both age and nutrition, whereas the chemical profile was only affected by age. In combination with previous studies, we conclude that wing fanning signals male quality and thus should be condition dependent, while the CHC profiles signal species identity and should remain stable. This further supports that *L. heterotoma* has a multimodal courtship display that conveys multiple messages. Additionally, we show that males increase their wing fanning frequency when mounted on the female. This behavioral adjustment during the courtship sequence is likely an energy-saving strategy, given the high energetic cost of wing fanning.

## Teaser

We show that in a parasitoid wasp, courtship components responded differently to male condition: Male wing fanning was affected by both age and nutrition, whereas a male’s chemical profile was only affected by age. In combination with previous results, we conclude that the chemical component is used mainly to identify species, while the vibratory component offers information on the condition of the male.

## Introduction

Sexual signals are widespread and play an important role in mate attraction and courtship. They often communicate essential information, such as species and sex of the sender (West-Eberhard 1983). Additionally, they can communicate a range of other traits that serve as quality indicators to the receiver of the courtship display (usually the female) (Candolin 2003). This can include traits, such as body size (Stoffer and Walker 2012), metabolic state (De Luca 2015), and age (Lassance and Löfstedt 2009; Nieberding et al. 2012), that allow the female to differentiate partner quality within a species. In order to communicate this information, animals use a wide array of sensory modalities. Because of our inherent human bias when observing animal behavior (Kokko 2017), there are some modalities that are more intuitive to us than others. Signal modalities widespread in arthropods but often overlooked are, eg vibrational signals (Cocroft and Rodríguez 2005; Hill and Wessel 2016) and chemical signals (Steiger and Stökl 2014).

Reflecting the variety of information that is communicated, most courtship displays are composed of multiple signals, often across different sensory modalities (Hebets and Papaj 2005; Mitoyen et al. 2019). This concept of multimodal communication can be traced back to Darwin (1871). However, research on signal evolution has long focused on single signals in isolation or signal specialists (Hebets and Papaj 2005). As multimodal signals can be more costly to produce and increase the risk of predation (Partan and Marler 1999), they raise interesting questions on the evolution of more complex signals over cheaper, simpler signals. This has resulted in a growing interest in multimodal signals in recent years. A variety of hypotheses have been proposed to explain the evolution of multimodal courtship despite its additional costs (Hebets and Papaj 2005; Wilson et al. 2013).

Broadly, hypotheses about multimodal courtship can be split into content-based and efficacy-based hypotheses (Guilford and Dawkins 1991). In efficacy-based hypotheses, the selection pressure is on the successful transmission of the signal, which can lead to redundant signals in different modalities (Hebets and Papaj 2005). For example, in the courtship of *Drosophila* males, which uses visual, acoustic, chemical, and tactile cues, the suppression of 1 modality does not alter copulation success (Colyott et al. 2016; Belkina et al. 2021). This suggests that in *Drosophila*, all 4 modalities redundantly contain the same signal. In contrast, in content-based hypotheses, the multimodal signal is used to transfer different information in the different modalities (Hebets and Papaj 2005). Content-based hypothesis can be further split into 2 categories: the species specificity hypothesis, where 1 modality communicates species identity and the other communicates quality, and the quality hypothesis, where the different modalities communicate different aspects of the sender's quality (Hebets and Papaj 2005).

Additionally, modalities within a multimodal signal might differ in how well they are suited to signal specific qualities. Thus, multimodal signals can not only contain information on different underlying qualities in different modalities, but their evolution might even be driven by these qualities (Higham and Hebets 2013). For example, in some birds, a diet rich in carotenoids can cause an accumulation of red pigments in their tissues (Price 2006). This red coloration, in turn, can serve as a reliable visual signal of a male's ability to acquire carotenoid-rich food, something that is more directly and effectively conveyed through color than through vocalizations. This correlation of quality and modality is well documented for visual signals (Hill and McGraw 2006), chemical signals (Steiger and Stökl 2014), and vibrational signals (Cocroft and Rodríguez 2005).

Life history theory predicts that traits under sexual selection tend to show heightened condition dependence and therefore provide more reliable signals of condition (Andersson 1982, 1986; Garland and Kelly 2006; Johnstone et al. 2009; Biernaskie et al. 2018). As the different modalities in a multimodal signal might face different amounts of selection pressure, different modalities could show different degrees of condition dependence. For example, in the complex visual and seismic courtship of the wolf spider *Schizocosa ocreata*, only some seismic traits are affected by male condition, implying that the visual signal has another function (Gibson and Uetz 2012). Despite extensive research on condition dependence in single signaling modalities, the role of condition in shaping multimodal signals has received little attention and remains an important open question.

In this study, we investigate condition dependence of the male courtship of *Leptopilina heterotoma*, a solitary larval parasitoid of *Drosophila* (Jenni 1951). Like many parasitoid wasps, *L. heterotoma* has a complex multimodal courtship display that consists of vibratory and chemical signals (Van Den Assem 1968). As females have to allow mating by exposing their genital structure and generally mate only once (Van Den Assem 1969), this system is shaped by female choice. Males begin their courtship by fanning their wings without flying while they follow the female around. They then mount the female and perform antennal tapping and additional bouts of wing fanning (Van Den Assem 1968). The antennal tapping is presumably to transfer an antennal pheromone (Isidoro et al. 1999; Weiss et al. 2015). This putative antennal pheromone is species specific and allows females to differentiate between 3 closely related *Leptopilina* species (Isidoro et al. 1999; Weiss et al. 2015). Wing fanning functions as a signal of male quality, as females preferentially mate with larger males exhibiting higher wing fanning frequencies (Lang et al. 2024). In contrast, the same study found no correlation between antennal pheromone composition and mating success. As females seem to be more selective about wing fanning than the antennal pheromone, we expect that wing fanning is under stronger sexual selection within a species, resulting in a stronger condition dependence (Andersson 1986). Previous research into the wing fanning of *L. heterotoma* has only focused on wing fanning while the male had mounted the female (Lang et al. 2024). In this study, we have also included analysis of the wing fanning that happens when males are not mounted on the female. We use diet manipulation and age differences to determine if these factors affect either of the 2 modalities in the courtship display of *L. heterotoma*.

## Materials and methods

### Study species

*Leptopilina heterotoma* is a koinobiont, solitary larval parasitoid of *Drosophila* with a large host range, including the model organism *Drosophila melanogaster* (Fleury et al. 2009). They mainly attack larvae in fermenting fruits and sap fluxes (Quicray et al. 2023). The female will lay eggs in the larvae, and if parasitism is successful, a wasp will emerge from the host pupae after around 20 d (Jenni 1951). Due to the aggregation of *Drosophila* larvae on single patches, *L. heterotoma* is quasi-gregarious. *L. heterotoma* show both on-patch and off-patch mating, resulting in a system of partial local mate competition (Hardy 1994; Fauvergue et al. 1999; Böttinger and Stökl 2020).

### Animal husbandry

Wild *L. heterotoma* were collected in Bayreuth in the summer of 2023. The strain was established in the lab and reared on *D. melanogaster*. Wasps and flies were kept at a constant 25 °C and about 60% humidity on a 16:8 h L:D cycle. The *Drosophila* were reared on a corn-based diet (1.9 L water, 150 g sugar beet syrup, 100 g cornmeal, 100 g wheat germ, 80 g yeast, 10 g agar, 5 mL propanoic acid, and 20 mL nipagin [10%]). Flies were placed on the medium for 2 d, and then removed. We then placed about 10 female and 3 male wasps in each tube (8 cm in height, 3.5 cm in diameter). Tubes were closed with foam plugs.

To ensure the wasps used in the experimental trials had not mated and were naïve, pupae were removed from the tube and individually placed in 1.5 mL microcentrifuge tubes. Each tube contained a piece of filter paper (0.5 cm diameter) soaked in 50:50 water and honey solution to feed the wasp after emergence. Tubes were checked for emergence of wasps once a day to determine days after emergence (henceforth referred to as age).

Females in both experiments were between 1 and 3 d old. In the “Age” experiment, young males were 1 to 3 d old and old males were 10 to 12 d old. For the “Nutrition” experiment, males were placed 1 d after emergence in a fresh microcentrifuge tube with filter paper either soaked in water (starved treatment) or 50:50 water and honey solution (fed treatment). In the "Nutrition" experiment, all males were tested 4 d after emergence. Experimental trials were randomized in both the “Age” experiment and the “Nutrition” experiment.

### Experiment

Single-choice mating trials were conducted in an arena made up of a Petri dish (4 cm in diameter). The bottom of the arena was a circular piece of printer paper, suspended between the 2 halves of the Petri dish to allow the paper to vibrate freely. The lid of the Petri dish was coated in a thin layer of Fluon (Antcube) and had a small hole (∼2 mm diameter) for the laser Doppler vibrometer (Polytech PDV-100, Waldbronn, Germany). Aligned with this hole, we glued a piece of reflectance foil onto the printer paper to amplify the laser-beam reflections. For each trial, 1 female was placed into the arena first, followed by 1 male. They were allowed to interact for 15 min, during which the laser recorded all vibrations. Additionally, each trial was recorded on video (Canon EOS 70D, 100 mm macro-objective). The video files were used to discriminate between wing fanning shown next to the female and wing fanning shown while the male had mounted the female. If either wasp tried to escape through the 2 mm hole in the arena, they were gently pushed back in with a paintbrush. If the wasp flew away before it could be pushed back into the arena or had to be pushed back 3 times or more, the trial was excluded (11/126 trials). This resulted in *N* = 57 trials in the “Age” experiment and *N* = 58 in the “Nutrition” experiment. After each experimental trial, wasps were frozen at −80 °C for chemical analysis and dissection.

### Body size analysis

To determine the size of each male used in our experiments, we measured the length of the tibia of the right hind leg. For this, the leg was dissected from freeze-killed wasps and mounted with adhesive tape on flash cards. Tibia length was then measured under a digital microscope (DvM6 M, Leica, Wetzlar, Germany) with 100× optical zoom. We found no differences between fed and starved males in the nutrition experiment (*t*(51.1) = −0.14, *P* = 0.889) or between old and young males in the age experiment (*t*(49.411) = 0.75, *P* = 0.459).

### Recording and analyzing vibrations

**Figure 1:**
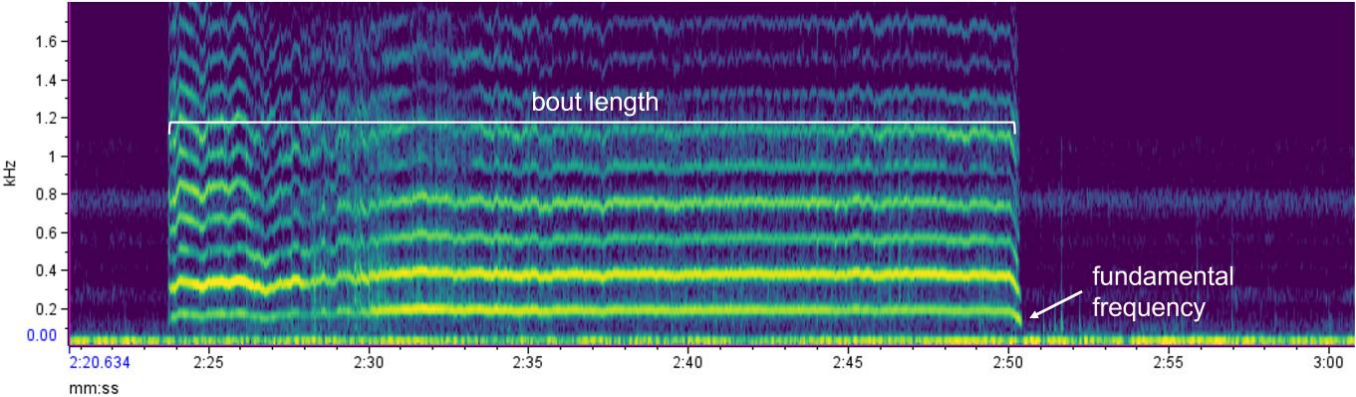
Example spectrogram of a wing fanning bout of a *L. heterotoma* male recorded with a laser Doppler vibrometer. For our analysis, we measured wing fanning bout length and fundamental frequency for each bout. The fundamental frequency corresponds to the wing fanning frequency of the wasp.

Any wing fanning that took place during the courtship trials was recorded with a laser Doppler vibrometer (Polytech PDV-100, Waldbronn, Germany). The vibrometer was set to a velocity measurement rate of 20 mm/s and a low-pass filter at 22 kHz. Recordings were done at a sampling rate of 44.1 kHz, using a laptop with a 64-bit sound card and Raven Pro v.1.6.5 sound editing software (K. Lisa Yang Center for Conservation Bioacoustics at the Cornell Lab of Ornithology, 2024). In each recording, all wing fanning bouts longer than 15 s were selected in Raven Pro v.1.6.5. In the nutrition experiment, we analyzed 2.55 ± 2.82 (mean ± SD, between 1 and 17 bouts) per male and in the age experiment, we analyzed 3.20 ± 2.44 (mean ± SD, between 1 and 11 bouts) per male. We analyzed wing fanning bout time (s) and fundamental frequency (Hz) (Fig. 1). Fundamental frequency was determined using the center frequency measurement in Raven Pro v.1.6.5 (the frequency that divides the selection into 2 frequency intervals of equal energy) while selecting only the first harmonic of each individual wing fanning event. This first harmonic is the wing fanning frequency (Webb et al. 1976; Bredlau et al. 2013). For ease of understanding, we will refer to the fundamental frequency as wing fanning frequency. We analyzed only trials in which wing fanning took place: *n* = 44 of 58 recordings in the “Nutrition” experiment and *n* = 45 of 57 recordings in the “Age” experiment.

### Chemical analysis

To extract their cuticular hydrocarbon (CHC) profiles, *L. heterotoma* males were allowed to thaw for 5 to 20 min. Then, they were extracted individually in 20 μL *n*-hexane for 10 min. Samples were analyzed in a gas chromatograph-mass spectrometer (GC2030 and QP2020NX, Shimadzu, Duisburg, Germany), with a nonpolar capillary column (SH Rxi-5 Sil MS, 30 m in length, 0.25 mm inner diameter, 0.25 μm film thickness, Shimadzu, Duisburg, Germany). Helium was used as the carrier gas (50 cm/s linear velocity). The oven program started at 80 °C, and temperature was raised by 5 °C/min to 280 °C. The putative male antennal pheromone of male *L. heterotoma* was first identified by Weiss et al. (2015). It consists of 22 CHCs, of which 9,19-pentatriacontadiene makes up around 50% of the pheromone (Weiss et al. 2015). We quantified all 22 CHCs with the *GCMS Postrun Analysis* software (Shimadzu, Duisburg, Germany). We created extracts of all males; however, we excluded runs that had CHC concentrations too low to be analyzed reliably (“Age” experiment, 1 of 57; “Nutrition” experiment, 21 of 58).

### Statistical analysis

We used R v. 4.3.2. for all statistical analyses (R Core Team 2023). *Dplyr* v.1.1.4 was used for data handling (Wickham et al. 2023), and plots were created using *ggplot2* (v.3.5.0) (Wickham et al. 2025). The data from the age experiment and the nutritional experiment were analyzed separately. For each experiment, we ran 2 generalized linear mixed models (GLMMs) fitted with the *glmmTMB* package (v.1.1.9.) (Brooks et al. 2025), evaluating effects on (ⅰ) male wing fanning frequency and (ⅱ) bout length (as response variables). Each model included the predictors’ condition and mounting state. Each model also included Trial ID as a random factor to exclude the effects of multiple bouts from the same trial/male. Subsequently, analyses of variance (ANOVAs) were conducted using the *car* package (v.3.1.3) (Fox et al. 2024). The models were checked using the *DHARMa* package (v.0.4.7) (Hartig et al. 2024). Scaled residuals were generated via simulation and tested for uniformity (Kolmogorov–Smirnov test), dispersion, zero inflation, and outliers. Residuals were visually inspected against fitted values and predictors to assess model fit and detect potential nonlinearity or heteroscedasticity (Hartig et al. 2024).

To compare the chemical profiles between groups of *L. heterotoma* males in both the “Age” and “Nutrition” experiments we used distance-based analysis based on centered log-ratio (clr) transformed data as described in Hervé et al. (2018). Data was clr transformed using the *compositions* package (v.2.0-9) (van den Boogaart et al. 2025), before we performed a nonmetric dimensional scaling (NMDS) ordination with Euclidian distance using the packages *vegan* (v.2.7-2) (Oksanen et al. 2025), *lattice* (v.0.22-7) (Sarkar et al. 2025), and *permute* (v.0.9-8) (Simpson et al. 2025) packages.

## Results

### Wing fanning

**Figure 2:**
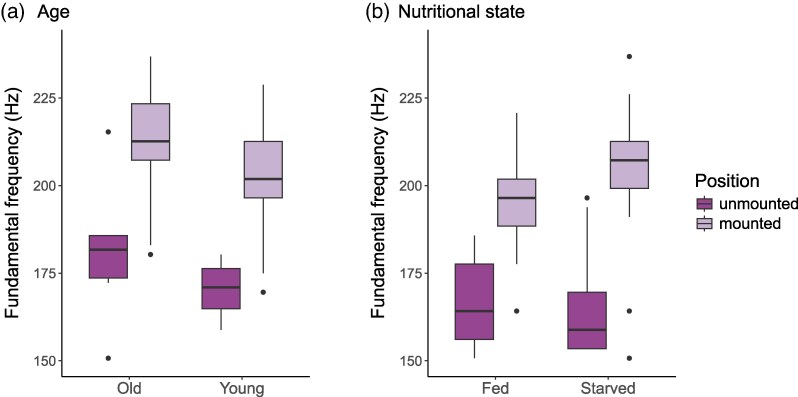
Boxplots showing the wing fanning frequency (in hertz) of a) old and young (*N* = 44) and b) fed and starved males (*N* = 45) of *L. heterotoma* when mounting a female and when positioned beside a female (unmounted).

Old males had a significantly higher wing fanning frequency than young males (Fig. 2; GLMM, χ^2^ (1) = 5.48, *P* = 0.019), and starved males had a higher wing fanning frequency than fed males (GLMM, χ^2^ (1) = 5.42, *P* = 0.020). Additionally, we found that males showed a significantly higher wing fanning frequency when mounted on the female in both the “Age” experiment (GLMM, χ^2^ (1) = 38.519, *P* < 0.0001) and the “Nutrition” experiment (GLMM, χ^2^ (1) = 115.81, *P* < 0.0001).

**Figure 3:**
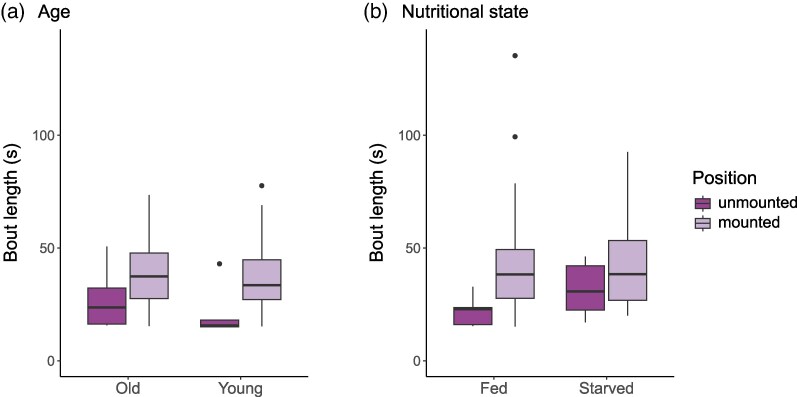
Boxplots showing the length of wing fanning bouts (in seconds) of male *L. heterotoma* in courtship trials of a) old and young (*N* = 44) and b) fed and starved males (*N* = 45) of *L. heterotoma* when mounting a female and when positioned beside a female (unmounted).

Wing fanning bout length was not affected by either age (GLMM, χ^2^ (1) = 0.18, *P* = 0.67) or nutritional status (GLMM, χ^2^ (1) = 0.0039, *P* = 0.95; Fig. 3). However, male wing fanning bouts were significantly longer when males were mounted on the female in both the “Age” experiment (GLMM, χ^2^ (1) = 8.68, *P* = 0.003) and the “Nutrition” experiment (GLMM, χ^2^ (1) = 6.04, *P* = 0.014).

### Chemical profiles

**Figure 4:**
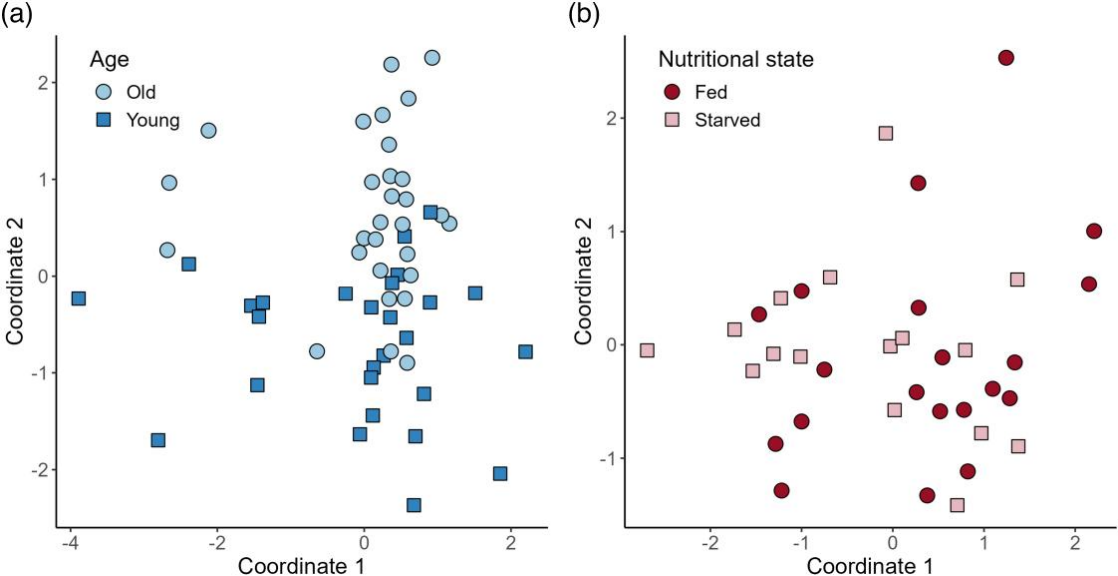
NMDS based on Euclidean distances of chemical profiles of *L. heterotoma* males of a) old and young (*N* = 56) and b) fed and starved males (*N* = 37) of *L. heterotoma*.

Statistical analysis of the putative antennal pheromone shows a significant difference between young and old *L. heterotoma* males (Fig. 4a; NMDS with clr stress = 0.14, ANOSIM with clr *R* = 0.24, *P* < 0.001, *N* = 56). Nutritional state had no effect on male putative antennal pheromone (Fig. 4b; NMDS with clr stress = 0.15, ANOSIM with clr *R* = −0.005, *P* = 0.47, *N* = 37).

## Discussion

In our study, male age was reflected in both wing fanning frequency and CHCs. Older males showed higher wing fanning frequencies than younger ones, and their CHC profiles differed. While wing fanning is a widespread courtship behavior among parasitoid wasps (Joyce et al. 2008; Danci et al. 2010; Bredlau et al. 2013; Canale et al. 2013; Benelli et al. 2014, 2020; Avila et al. 2017; Zeni et al. 2024), this is the first study to show the condition dependence of wing fanning in a parasitoid wasp. However, there is evidence that similar courtship behaviors are condition dependent, eg the courtship vigor of male *Drosophila grimshawi* is affected by adult diet (Droney 1998). Similarly, in *Gryllus campestris*, males in a better condition call more frequently and attract more females (Holzer 2003). Vibrational signals in a nuptial gift-giving spider are also affected by condition (Eberhard et al. 2020), as well as the vibrational signal of wolf spiders (Lomborg and Toft 2009).

As we know that *L. heterotoma* males with a higher wing fanning frequency are more likely to mate (Lang et al. 2024), this would mean that older males are more attractive than younger males. A similar preference is seen in the acoustic courtship of *G. campestris*, where older males’ calling songs are more likely to attract females, with more pulses per chirp and a lower carrier frequency (Scheuber et al. 2004; Jacot et al. 2007). Similarly, insects can also communicate their age with chemical signals. For example, in both the butterfly *Bicyclus anynana* and the burying beetle *Nicrophorus vespilloides*, the composition of the male sex pheromone changes with age, and females prefer the older males (Nieberding et al. 2012; Chemnitz et al. 2015). This preference for older males is not limited to invertebrates. For example, in guppies, male ornamentation increases with age and in turn makes older males more attractive than younger males (Miller and Brooks 2005).

There are several hypotheses to explain why older males might be more attractive. One possibility is that males that have lived longer have already been subject to viability selection, such that lower-quality individuals are more likely to have been removed from the population over time (Manning 1985). As a result, older males may represent a subset of higher-quality individuals. Although males in this study were laboratory reared, precluding natural viability selection, females may still use age as a cue for variation in signaling performance. In addition, age may signal heritable viability, such that females mating with older males could obtain indirect genetic benefits if longevity has a genetic component (Manning 1985; Kokko 1998; Brooks and Kemp 2001). Additionally, age-based advantages may arise through experience or physiological effects of repeated activity; eg repeated use of the flight musculature over time may improve the efficiency or coordination of wing fanning displays, potentially enhancing signaling performance. This could reflect general use-dependent maintenance or plasticity of flight muscle performance, as insect flight muscle function can be influenced by activity levels (Marden 2000), and behavioral performance in insects can improve with experience (Dukas 2004). This is consistent with models of age-dependent sexual advertisement and terminal investment, which predict increased allocation to costly courtship displays as future reproductive opportunities decline (Williams 1966; Clutton-Brock 1984; Kokko 1998; Proulx et al. 2002).

While CHC profiles also varied with age in our study, there is no evidence that females of *L. heterotoma* prefer a certain chemical composition of the male sex pheromone (Lang et al. 2024). While it is possible that there is a female preference that the previous study was not able to detect, there are also cases in which a signal is condition dependent, but it is not used by the females to discern male condition. For example, the chemical signal of *Drosophila simulans* males changes with diet manipulation, but this does not impact their attractiveness to females (Ingleby et al. 2013). Similarly, in *Mantophasmatodea* heelwalkers, vibrational signals vary with condition and age, but females do not differentiate between vibrational signals of males with different conditions or ages (Eberhard et al. 2019).

In our diet manipulation experiment, we found a difference in wing fanning, but no effect of nutritional state on CHCs. Unexpectedly, starved males showed significantly higher wing fanning frequencies than fed males. Typically, motor-skill or vigor-based signals, such as wing fanning, are seen as an honest signal of whole-organism quality (Byers et al. 2010). As they are energetically costly, most studies show that food limitation results in a lower reproductive effort (Duffield et al. 2017). One example of this are wolf spiders, where male condition improved dynamic signal attributes, such as the amplitude and rate of cheliceral strikes and the rate of double tapping in their seismic courtship signal (Gibson and Uetz 2012). However, there are also some examples where nutritional stress leads to increased courtship signaling. Examples for this are food-deprived stickleback fish, who show brighter coloration (Candolin 1999), or parasitized *Drosophila* males that increase their courtship activity (Polak and Starmer 1998). We thus interpret the higher wing fanning frequency of starved *Leptopilina* males as an increased courtship effort, indicating a terminal investment strategy.

Frequency is often also correlated with size, as larger animals typically are able to produce lower-frequency sounds (Rodríguez et al. 2015). However, as male size did not differ between the different treatment groups in our study, this seems unlikely. This is not unusual in Hymenoptera, as, eg Conrad et al (2010) showed that male vibrations differed between males accepted for copulation by females and those who were not. However, they also showed that size did not predict mating success.

In contrast, we saw no effect of food availability on the composition of male CHCs. While effects of food quantity and diet quality on CHC seem widespread in insects and are well documented (Otte et al. 2018), this fits into previous findings in *L. heterotoma*, where no distinct differences in CHC patterns were observed between successful and unsuccessful males (Lang et al. 2024). Additionally, we know that CHCs are species specific and used for species recognition in *L. heterotoma* (Weiss et al. 2015). This could result in CHC composition being under stabilizing selection to maintain a reliable signal of species identity. Consistent with this, CHC profiles of sympatric species appear more strongly differentiated in multivariate space than the within-species variation observed here (Weiss et al. 2015), suggesting that CHCs function as species recognition cues.

We have shown that the different modalities in the courtship of *L. heterotoma* are affected differently by male age and nutritional status. This suggests that the different courtship modalities are used to communicate different information, thus supporting the multiple messages hypothesis (Hebets and Papaj 2005). Females choose males based on their wing fanning performance (Lang et al. 2024). Indeed, we find that wing fanning is affected by both age and nutrition, making it a reliable indicator of male condition. Females do not choose males based on their chemical profile (Lang et al. 2024), but they reject heterospecific males that have a distinct chemical profile (Weiss et al. 2015). This suggests that the chemical modality is a species-specific signal to convey species identity, but not male quality. This fits with our result that the CHC profile was not affected by male nutrition. However, we did find an effect of age on male CHCs.

If a multimodal courtship signal is used to communicate multiple messages, differing degrees of (sexual) selection pressure on different signals in different modalities may favor the evolution of such multimodal signaling. Signals conveying species identity (eg male CHCs) should remain relatively stable and independent of the sender's condition to fulfill this function, whereas motor performance traits (eg wing fanning) can indicate vigor only if variation in performance reflects differences in male condition (Byers et al. 2010). Thus, communicating different traits may require different levels of plasticity.

Information can also vary within a modality across time and space (Uy and Safran 2013). For example, in the field cricket (*Teleogryllus oceanicus*), acoustic signals are used for both long- and short-range communication (Zuk et al. 2008). So, when looking at multimodal displays, it ‘s important to look not only at the different modalities but also at variation within each modality. Indeed, we found that across all treatment groups, males showed a higher wing fanning frequency and longer bouts when they were mounted on the female than when they were not mounted. A similar phenomenon has been found in *Psyttalia concolor*, another parasitoid wasp with a similar courtship sequence (Benelli et al. 2012). However, the frequency difference in *P. concolor* is only 4 Hz, while in *L. heterotoma*, we found a much larger increase (between 32 and 49 Hz) in mounted female bouts. In the study that investigated *L. heterotoma* courtship previously, only wing fanning on the female was measured, as it is much more obviously linked to subsequent copulation or rejection by the female (Lang et al. 2024). The fact that males adjust their wing fanning effort at all seems to indicate that wing fanning on and off the female are distinctly different behaviors that might serve different functions. Generally, lower-frequency vibrational signals propagate further along plant matter (Cocroft and Rodríguez 2005). Therefore, unmounted wing fanning might be more likely to reach the female at a lower frequency. Wing fanning at the high frequencies shown by these hymenopteran requires extremely specialized high-frequency flight muscles and places high metabolic demands on the individual (Beenakkers et al. 1984; Hickey et al. 2022). It is therefore plausible that the higher-frequency male display on the female is energetically costly to the male and could signal male condition. However, we do not know why males show wing fanning when not mounted on the female, despite this behavior pattern being widespread in the courtship behavior of parasitoid wasps (Van Den Assem 1969; Benelli et al. 2012; Avila et al. 2017; Benelli et al. 2020; Zeni et al. 2024).

Overall, this study provides further insight into the information content of the multimodal courtship signal in *L. heterotoma*. We show that wing fanning may communicate male age and nutritional state and that male chemical profiles change with age. Given that chemical signals are known to convey species identity (Weiss et al. 2015), our results provide additional evidence that the multimodal courtship of *L. heterotoma* transmits conveys multiple, functionally distinct messages. This makes *L. heterotoma* one of only very few species where the information content in more than 1 modality has been analyzed in detail (Hebets and Papaj 2005; Mitoyen et al. 2019).

## Data Availability

Analysis reported in this article can be reproduced using the data provided by Plate et al. (2026).
